# An Ishmaelite

**Published:** 1888-08-04

**Authors:** Leslie Keith

**Affiliations:** Author of "The Chilcotes," "Uncle Bob's Niece," "East and West," etc.


*(To be continued.)*

### AN ISHMAELITE.

BY LESLIE KEITH,

Author of "The Chilcotes," "Uncle Bob's Niece," "East and West," etc.

*(Continued from p. 283.)*

### CHAPTER XVII.—A HAPPY RELEASE.

ELLA had got home, having this time made the journey without Arthur's society. She had travelled in the company of a young village girl who had been selected to be trained for service in Portland Place, and, as far as Arthur knew, this little maid was her only companion. But when he went to the station to meet her, he was surprised to see Ella shaking hands with a short stout man whose back was towards him, but whose figure seemed somehow remotely familiar.

Ella greeted him with something less than her usual composure.

"I did not expect you, Arthur," she said, "you need not have taken the trouble to come all this way."

"You don't seem to have missed me," said Arthur good humouredly. "Was that Penfold who left you just now?"

"Yes," said Ella, exhibiting some small signs of confusion, "he had to come to town on business, and we travelled together."

Arthur laughed. The Penfold scheme and Ella's maidenly objections to touch upon it, amused him, and yet the fact of the two having journeyed together seemed to give colour to cousin Fanny's hopes.

"Has he got a country house to his mind?" he asked, when they were seated in the cab. "Your letters told me nothing, Ella. And is he meditating matrimony? How is Clara, by-the-by, and my little friend Lettie?"

"Lettie is quite well," said Ella almost pettishly; "and Arthur, if you don't mind, I'd rather not talk. I am tired, and I have to speak so loud to make you hear."

He acquiesced in her wish for silence, but he felt there was something wrong about their meeting. Two young folks who are lovers as well, ought not to have found any supreme difficulty in talking to each other after a separation of two months, even if the traffic of London made it necessary to raise the voice.

They ought to have had a good deal to say to each other, and they had nothing. Even when they had got back to Portland Place, when Ella had shaken hands with cousin Joseph, and had washed off the stains of travel, and was seated in her accustomed place in the drawing-room, she seemed in no haste to communicate her news.

Ella had so frequently quoted "Papa and Mamma" to Arthur, that he found her reticence odd. She puzzled him altogether. There were differences in her which he felt, though they escaped his analysis. At one moment he fancied she was troubled, at the next offended, but he knew of nothing in himself to give him any clue to her altered mood.

In the months when they had been separated, Arthur had done a good deal of repentance. He knew that he had been chiefly in fault, and like an honest man he took himself to task, and pledged himself to honour and respect the woman he had loved well enough to choose as his wife. "She has not changed," he said, which was no doubt true enough; it was only his own standpoint that had altered. Ella was just the same, but Arthur looked at her with different eyes. However, in his new humility he was very kind to her, and gentle; all his best resolutions were hot in him; except on the one point, of seeing Fred, he would yield to her in everything.

"If I don't love her as much as I thought, I must make it up to her in other ways," and the compensation Ella would appreciate most was obedience to her authority. "She will prize it more than my love," thought poor Arthur, and he proceeded to practise his virtuous intentions by wiping his feet conscientiously on the mat, dressing himself carefully for dinner, and sitting throughout that solemn ceremony without impatience. He had done everything Ella liked him to do, and yet she remained unresponsive.

The explanation was given in a day or two. When Arthur came home earlier than usual one afternoon, he ran against Mr. Penfold, being at the moment ushered out by the butler.

Arthur renewed his brief acquaintance with this gentleman, suppressing a smile as he recalled the artless Miss Lettie's revelations, but the departing guest declined his invitation to return and smoke, and seemed, indeed, in a great hurry to get away.

Still the unsuspecting Arthur guessed nothing, and put the visit, if he thought of it at all, down to civility, or the desire to be near Clara's sister, since Clara herself was beyond his reach. Arthur had been in love himself, and could follow the phases of the malady, even when the sufferer was an elderly gentleman. Even when Ella sent for him, he remained unenlightened.

Ella was standing near the window of the drawing-room, but with her back to it. She did not move when he went up to her with a careless "Well Ella, here I am;" but her agitation subtly conveyed itself to him.

"What is it?" he said more seriously; "what has happened?"

"Mr. Penfold has been here," she said.

"I met him," said Arthur, looking rather surprised.

"Do you—do you know why he came, Arthur?"

"No," he said, feeling bewildered, "unless it was about Clara ——"

"He asked me to marry him."

Arthur stared at her, her words were spoken in so low a tone that he scarcely took in their sound at first, and it was still longer before their meaning dawned on him.

"Do you want to marry him, Ella?" he asked at last.

She looked down, and blushed, and twisted the cord of the window-blind in her fingers—a sign of great perturbation on the part of the composed young woman.

"Do you love me, Arthur?" she said. "For a long time you have ceased to care for me; and though you have tried to hide it, I have felt that you were not the same. You refused to come to us in the country, you left me for two months."

"And another has stepped into my place," said Arthur; and he laughed out loudly, but without mirth: "So it wasn't poor Clara, after all, in spite of the new dress, and the new furniture, and the late dinner."

"Arthur," said Ella, recovering some of her dignity, "I told Mr. Penfold that I was pledged to you, and that I could not listen to him until ——"

"Until I release you. I do so now, Ella," said Arthur, speaking gravely and rather sadly. "Why should I hold you to your word when your heart no longer goes with it."

"I don't think you ever gave me yours, Arthur."

"Perhaps not," he said gently. "Why should we dispute about it? Let us be friends, Ella, though we are to be lovers no longer. I wish you happiness with all my heart, and a better husband than I."

"I think I can make Mr. Penfold happy; he is very kind, and he doesn't expect too much."

There was a little sting in the words, perhaps. Though Ella was the defaulter she did not care to be relinquished so readily; and indeed, when Arthur came to think matters over, he was ashamed to reflect how easy it had been for him to yield his claims to the new aspirant.

"You will find someone else who will understand you better," she had said, and he did not deny it. Perhaps he had found the someone else already, though he did not know it, and though he had honestly meant to keep his compact and be a kind and loyal husband.

When he came to know the favoured lover better, he acknowledged that Ella had chosen wisely for herself. Mr. Penfold was a very simple gentleman, and as he had an honest conviction that Ella could do no wrong, their matrimonial lines were likely enough to run smooth. The only pity was that he could not marry Clara as well, and thus reward poor Cousin Fanny, whose sacrifices had all gone for nothing.

"I will tell Cousin Joseph myself," said Ella, "since it is I who have taken this step;" and when Arthur would have helped her in this unpleasant task, and would have taken the onus of the change on his own shoulders, she refused him firmly.

"Perhaps he will not wish me to stay here any more," she said.

"Why shouldn't he? There never was a better mistress," said Arthur heartily. He had an idea that she might not get a very warm welcome at the vicarage, where, no doubt, there was some soreness of feeling over Clara's defeat, and he determined to espouse her cause with his uncle. It was very easy to be friendly and kind now that he was expected to be nothing more.

Ella found it less difficult to break the ground with Mr. Eastgate than it had been with Arthur. Cousin Joseph was sensible, and he did not bewilder her with outbursts of sarcasm or bitterness. He listened in a grim silence while she told her tale.

"Arthur never really cared for me," she repeated, anxious to justify herself in his eyes. "He put others before me. He insisted that I should go abroad with him ——"

"Go abroad with him?" demanded Mr. Eastgate, looking at her from under his grey, bushy brows. "What do you mean?"

"He wanted to settle abroad, in one of the colonies, because of—your son ——"

A slight shock seemed to pass over the listener, but he drew himself together and said briefly—

"Go on."

"He thinks of Fred before anyone else," said Ella, taking courage from his quietness, and letting loose her long-pent-up grievance. She knew that it was a law of the house not to mention Fred, but she did not see why Cousin Joseph should not know how much she had had to suffer. She had given Arthur up, and perhaps that was why she found it so difficult to forgive him.

"It is always Fred; he thinks of no one else," she repeated. "He goes there every night—you must have noticed how much he is away ——"

Mr. Eastgate did not deny the assertion. He sat staring at her with a curious look—if Ella had been quicker at deciphering expression, she might have called it a hungry look—but his lips only uttered one word—

"Where?"

She easily enough told him all she knew, which was not much.

"Down in some low part of Bermondsey—Gillespie Street, I believe; he lives with some quite poor, common people; a stationer's shop, I think Arthur said it was. He wanted me to go, but of course that was out of the question. A lady can't go to such places, as Arthur might have known ——"

She had been all the while occupied with her own grievance, and for a young woman of her tact had certainly blundered in not considering how her words might affect the listener. His silence seemed to strike her at last.

"I am sure, cousin Joseph," she said, "I am very sorry if I have hurt or offended you, but really you know ——"

Her pause made him look up. He was not thinking of her or her feelings, or her trespasses against good taste. He was thinking of the stationer's shop in Gillespie Street, Bermondsey.

"You had better ask Penfold to dinner," he growled, and Ella understood that she was to remain in Portland Place until Mr. Penfold took her to preside over his own establishment.

She was very well content with this arrangement, since it would not have been pleasant to go home again so soon, and she was more than ever sure that she had done right in taking Mr. Eastgate into her confidence when that same evening Arthur slipped out of the house immediately after dinner. Surely cousin Joseph must feel now that she was justified in giving him up.

Mr. Eastgate took no notice of his absence, but when Mr. Penfold had eaten the dinner to which, as Ella's accepted suitor, he was bidden, and had taken his departure, he remarked to Arthur—

"So that fellow has ousted you?"

"So it seems."

"You don't break your heart over it."

"He's a very decent old buffer," said Arthur with easy generosity, "and I don't grudge Ella a far better husband than she would have had in me. I think she has chosen wisely for her own happiness."

"Perhaps you will find a better wife than she would have made."

That was Mr. Eastgate's only comment on Ella's new disposal of her affections, but, considering that she was an Eastgate on her mother's side, it was surely a tremendous admission for cousin Joseph to make.

